# Associated factors related to chronic kidney disease progression in elderly patients

**DOI:** 10.1371/journal.pone.0219956

**Published:** 2019-07-23

**Authors:** Cláudia Tótoli, Aluizio Barbosa Carvalho, Adriano Luiz Ammirati, Sergio Antônio Draibe, Maria Eugênia F. Canziani

**Affiliations:** Department of Medicine, Division of Nephrology, Federal University of São Paulo, São Paulo, Brazil; University of Sao Paulo Medical School, BRAZIL

## Abstract

**Background:**

Chronic Kidney Disease (CKD) is a worldwide public health problem. The prevalence of CKD is rising especially in elderly, as consequence of population-ageing related to socioeconomic development and better life expectancy. There are scarce studies evaluating CKD progression and its associated factors in elderly patients.

**Methods:**

This is a retrospective observational study including 340 patients (≥ 65 years old) CKD stages 3a–5 non-dialysis, incidents in an outpatient CKD clinic, followed by 2.1 years. CKD progression was assessed by the slope of eGFR calculated by CKD-EPI and BIS 1 equations. The patients were divided in progressor and non-progressor groups (eGFR slope < or ≥ 0 mL/min/1.73 m^2^/year, respectively).

**Results:**

Kidney function declined in 193 (57%) patients. In this group, the progression rate was -2.83 (-5.1 / -1.1) mL /min /1.73 m^2^ /year. Compared to non progressor, the progressor patients were younger [72 (69–78) vs. 76 (69–80) years; p = 0.02]; had higher proportion of diabetic nephropathy, higher serum phosphorus [3.8 (3.3–4.1) vs. 3.5 (3.9–4.1) mg/dL; p = 0.04] and proteinuria [0.10 (0–0.9 vs. 0 (0–0.3)] g/L; p = 0.007)] at the admission. In the logistic regression analysis adjusted for gender and eGFR, proteinuria was independently associated with CKD progression [OR (Odds Ratio) (1.83; 95% CI, 1.17–2.86; p < 0.01)].

**Conclusion:**

CKD progression was observed in the majority of elderly CKD patients and proteinuria was the most important factor associated to the decline of kidney function in this population.

## Introduction

Chronic Kidney Disease (CKD) is a worldwide public health problem. CKD is one of the leading causes of non-communicable diseases and its prevalence is rising especially among older adults, as a consequence of socioeconomic development and better life expectancy [1].

The estimated glomerular filtration rate (eGFR) in elderly people has been a challenge. The 2012 Clinical Practice Guidelines KDIGO [2] has recommended CKD Epidemiology Collaboration equation (CKD-EPI), despite only a few number of older adults have been included in the cohort that validated this equation. In the same year, two other formulas, the Berlin Initiative Study (BIS)-1, based on serum creatinine and the BIS-2, based on serum creatinine and cystatin [3], were suggested as the preferable methods to estimate GFR in older people. However, subsequent reports did not confirm the superiority of BIS over CKD-EPI to diagnose CKD in this population [4, 5].

Beyond diagnose, the progression of CKD in elderly people has been an additional task. As aging has been associated to kidney dysfunction, the differentiation between CKD progression, per se, and the age-related decreasing of GFR has been a matter of discussion [6]. Additionally, many definitions of CKD progression have been used over the years, such as: doubling of serum creatinine [7], eGFR decreasing > 15 mL/min/1.73 m^2^ and achievement of end stage renal disease (ESRD) [8]. In studies comprising elderly CKD populations, the changing of eGFR in mL/min/year has been the most frequently parameter used, albeit with conflicting results. In observational studies, while some authors have shown that elderly people present a slow or none decline of kidney function [9, 10], others reported that the rate of eGFR lost could be faster [11].

Based on the scarce and conflicting fore mentioned data, we aimed to describe the associated factors and the behavior of the CKD progression in a cohort of elderly patients.

## Methodology

### Population

All incident CKD stage 3-5ND patients from the outpatient CKD Clinic of the Federal University of São Paulo, Brazil, from April 2011 to April 2015, were evaluated. Among 1319 patients, 663 were ≥ 65 years. Exclusion criteria comprised: follow-up time shorter than one year (n = 218) or only one serum creatinine measurement (n = 105). Therefore, 340 elderly patients were included in the study.

The study was reviewed and approved by the Ethics Advisory Committee of the Federal University of São Paulo (approval number: 0912/2015, CAAE: 47507215.6.0000.5505) and the need for informed consent was waived. All data were anonymized to comply with the provisions of personal data protection legislation.

### Study design and protocol

This was a single center retrospective observational study. All the demographic, clinical and laboratorial data at the first visit (admission) were obtained from patients´ files. Laboratory evaluation included: urea, hemoglobin, sodium, potassium, ionized calcium, phosphorus, intact parathyroid hormone, bicarbonate, glucose, glycated hemoglobin, uric acid, low- (LDL) and high-density lipoprotein (HDL) cholesterol and triglycerides. Proteinuria was assessed in spot urine sample and classified as absent, mild (>0 to 0.99 g/L), moderate (≥1 to < 3 g/L) or severe (≥3g/L). The creatinine of the admission and the last one available in the patients’ files were used to calculate eGFR (CKD-EPI) 1 and 2, respectively. CKD progression was assessed by the rate of eGFR slope over time [(eGFR 2 –eGFR1)/ follow up (yr)]. The patients were classified into ‘progressor’ and ‘non-progressor’ groups (eGFR slope < or ≥ 0 mL/min/1.73 m^2^/year, respectively). Patients with slope < - 5 mL/min/1.73 m^2^/year were considered as fast progressors. The BIS 1 equation was also determined. Hypertension was defined as blood pressure > 140/90mmHg or use of any anti-hypertensive drug; anemia as hemoglobin < 10 mg/dL; and obesity and overweight as body mass index (BMI) ≥ 30 and 25 Kg/m^2^, respectively.

### Statistical analysis

Mean and standard deviation, median and interquartile range or frequencies (proportion) were calculated for each variable, as appropriate. The Kolmogorov-Smirnov statistical test was used to investigate the variable distribution. Comparisons of continuous variables were performed using Student’s t-test and the Mann-Whitney U-test, for normal and skewed data, respectively. Comparisons of proportions were performed using chi-squared analysis or Fisher’s exact test, as appropriate. Multiple imputations were performed for missing data using MICE package of software R. This approach was applied only to variables with up to 25% missing data. Thus, all the patients were included for the logistic regression analysis. A stepwise multivariate model was constructed to test the variables associated with CKD progression. This method selected variables with p < 0.10, adjusted for gender and CKD-EPI at the admission. Cox proportional hazards model was used to investigate the impact of the occurrence of death on the CKD progression. Values of p < 0.05 were considered statistically significant. All analyses were performed using a standard statistical package (SPSS for Windows, version 20; SPSS, Chicago, IL, USA).

## Results

**Fig 1:**
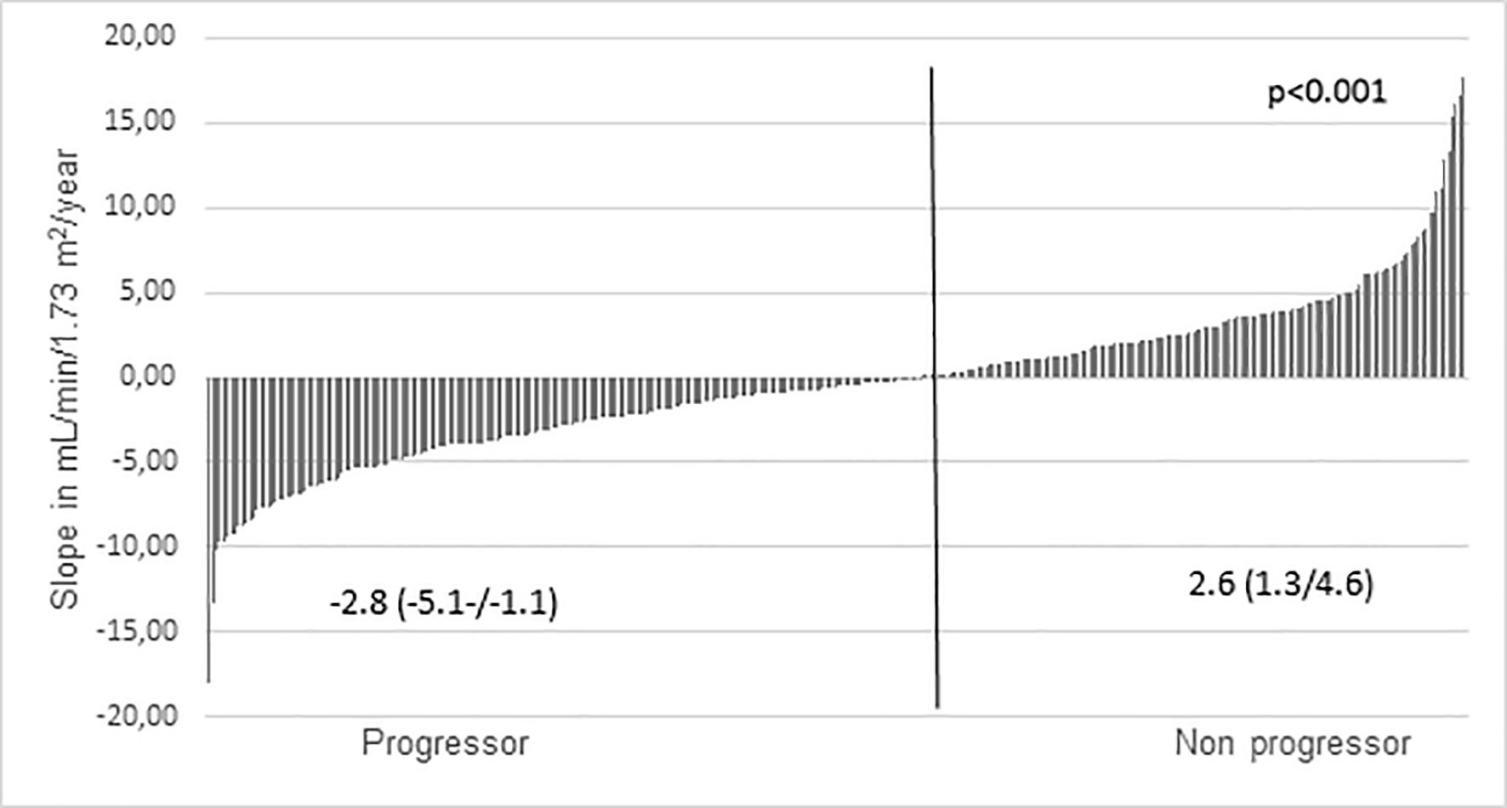
Cohort classification according individual eGFR slope during the study eGFR slope of each patient during the study and cohort classification.

Table 1 depicts the patient’s characteristics at the admission. Patients were predominantly male, white, and CKD stages 3a (11.5%), 3b (37%), 4 (43%) and 5 (9%). Diabetic nephropathy and hypertension were the main cause of CKD. Hypertension was present in 62% and anemia in 11% of the patients. Proteinuria was observed in 46% of the population. The prevalence of obesity and overweight were 28% and 40%, respectively. The patients were followed for 2.1 (1.5–3.1) years. Of all, 12 (3.5%) patients have died, 53 (16%) withdrawal treatment and 20 (6%) required dialysis during the follow up period. The median eGFR 1 was 29.1 (20.9–39.0) and eGFR 2 was 27.9 (18.7–38.2) mL/min/1.73m^2^. During the follow up eGFR declined in 193 (57%) patients (progressor group, Fig 1)

**Table 1:** Characteristics of all patients and comparison of demographic, clinical and laboratory parameters inter group.

	n	All	Progressor n = 193	Non progressor n = 147	p
Age (years)	340	73 (69–79)	72 (69–78)	76 (69–80)	0.02
Male n (%)	340	191 (56)	105 (54)	86 (58)	0.45
Caucasian n (%)	298	194 (65)	114 (67)	80 (62)	0.34
Etiology n (%)	340				0.04
Hypertension		63 (19)	29 (15)	34 (24)	
Diabetes mellitus		94 (28)	62(32)	32 (22)	
Others		183 (54)	102 (53)	81 (55)	
iACE/ARB use(%)^a^	340	227 (67)	135 (70)	92 (62)	0.15
Body mass index (Kg/m^2^)	238	27.1 (24.2–30.4)	27.0 (24.0–30.4)	27.4 (24.6–30.0)	0.98
Systolic Blood Pressure (mmHg)	339	140 (130–160)	140 (130–160)	140 (120–150)	0.12
Diastolic Blood Pressure (mmHg)	339	80 (80–90)	80 (80–90)	80 (72–90)	0.07
Creatinine (mg/dL)	340	1.94 (1.60–2.57)	1.92 (1.52–2.58)	2.00 (1.67–2.46)	0.13
Ureia (mg/dL)	319	80 (63–106)	79 (62–102)	80 (64–108)	0.85
Proteinuria (g/L)	286	0.00 (0.00–0.50)	0.10 (0.00–0.90)	0.00 (0.00–0.30)	0.007
Sodium (mmol/L)	295	140 (138–142)	140 (138–142)	140 (138–142)	0.31
Potassium (mmol/L)	314	4.8 (4.4–5.2)	4.8 (4.4–5.3)	4.7 (4.4–5.2)	0.27
Hemoglobin (g/dL)	299	12.4 ±2.0	12.4 ± 2.0	12.4 ± 2.1	0.88
Ionic Calcium (mmol/L)	130	1.27 ± 0.08	1.28 ± 0.08	1.26 ± 0.09	0.35
Phosphorus (mg/dL)	180	3.7 (3.2–4.1)	3.8 (3.3–4.1)	3.5 (2.9–4.1)	0.04
Parathyroid hormone (pg/mL)	71	124 (79–197)	133 (94–189)	95 (53–226)	0.28
Bicarbonate (mmol/L)	129	25.17 ± 3.63	25.44 ± 3.33	24.84 ± 3.98	0.35
Fasting plasma glucose (mg/dL)	269	104 (91–120)	104 (90–122)	105 (97–114)	0.74
Glycated hemoglobin (A1C) (%)^b^	142	7.1 (6.4–8.4)	7.4 (6.4–8.6)	7.0 (6.4–8.3)	0.34
LDL cholesterol (mg/dL)	278	104 (81–130)	105 (81–135)	103 (81–122)	0.52
HDL cholesterol (mg/dL)	227	44 (35–55)	44 (35–55)	43 (35–55)	0.76
Triglycerides (mg/dL)	224	132 (94–177)	139 (93–192)	129 (95–173)	0.51
Uric acid (mg/dL)	88	7.6 ± 1.82	7.7 ± 1.54	7.6 ± 2.14	0.71

a—angiotensin-converting-enzyme inhibitors or angiotensin-receptor blockers

b- only diabetes patients

**Fig 2:**
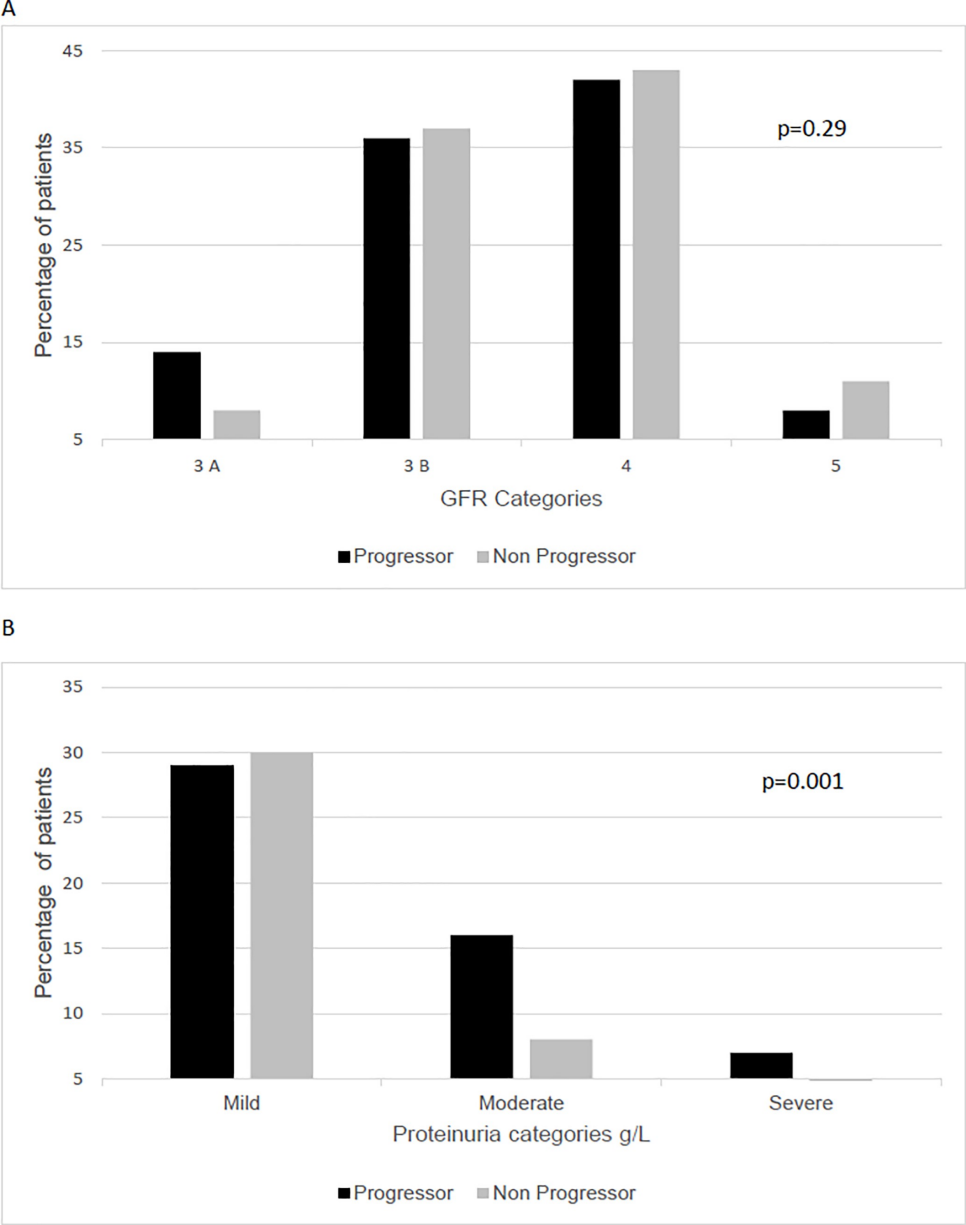
A Distribution of the patients according to CKD stages at baseline Fig 2. B Distribution of the patients according to proteinuria at baseline.

**Fig 3:**
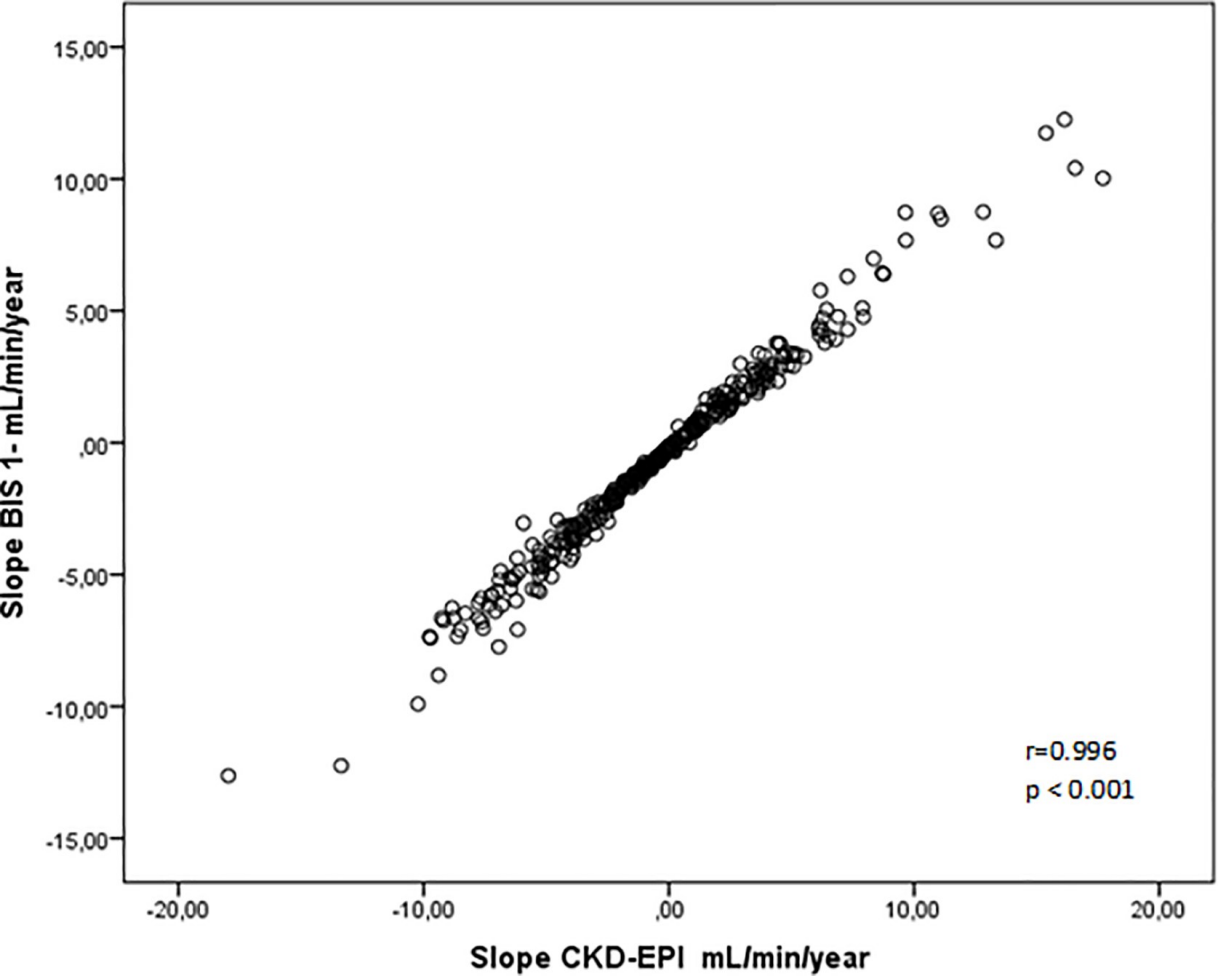
Correlation between BIS-1 slope and CKD-EPI slope.

The characteristics of the progressor and non progressor groups were showed in Table 1. Comparing to non progressors, the progressors patients were younger, had higher proportion of diabetic nephropathy and had higher proteinuria and serum phosphorus. There was a trend to a higher diastolic blood pressure in the progressor group. The distribution of the patients according to CKD stages and proteinuria categories were shown in Fig 2A and 2B. CKD stages were similar in both groups (Fig 2A), and the proportion of patients with moderate and severe proteinuria was significantly higher in the progressor group (p = 0.001, Fig 2B). There were no differences regarding the use of angiotensin-converting-enzyme (ACE) inhibitors or angiotensin-receptor blockers (ARBs). In the logistic regression analysis, only proteinuria was independently associated with CKD progression [OR (Odds Ratio) (1.83; 95% CI, 1.17–2.86; p < 0.01)]. In the Cox proportional-hazards model, the occurrence of death was not associated to CKD progression [HR 0.54 (CI 0.17–1.70)]. Table 2 shows the comparison between eGFR data and slope, based on the CKD-EPI and BIS 1 equations. No significant difference in the measurements of eGFR1 between groups either based on CKD-EPI or BIS 1 equations. As expected, eGFR2 was significantly lower and higher than eGFR1, in progressor and non progressor groups, respectively. Fig 3 depicts the slopes, calculated by both equations (r = 0.99, p<0.001).

**Table 2:** Comparison of renal function and progression parameters inter groups.

	Progressor (n = 193)			P^a^	Non Progressor (n = 147)			P^b^	Inter groups	
	eGFR 1	eGFR 2	Slope		eGFR 1	eGFR 2	Slope		P^E^	P^U^
**CKD-EPI(mL/min/1.73m**^**2**^**)**	30.1 (21.5/39.3)	22.0 (14.8/31.8)	-2.8 (-5.1/ -1.1)	<0.001	29.0 (20.5/36.7)	37.6 (27.7/44.9)	2.6 (1.3/4.6)	**<0.001**	**0.28**	**<0.001**
**BIS (mL/min/1.73m**^**2**^**)**	32.6 (25.8/39.7)	25.9 (19.8/33.5)	-2.5 (-4.4/ -1.1)	<0.001	31.4 (23.1/36.8)	36.8 (29.3/43.1)	1.8 (0.8/3.3)	**<0.001**	**0.08**	**<0.001**

p^a^ = p intra group progressor; p^b^ = intra group non progressor; p^E^ = p intergroups admission; p^u^ = p intergroup last creatinine available

In the progressor group, 49 out of 193 (25%) patients were fast progressors and their median eGFR slope was -6.9 (-8.4 / -5.6) mL/min/1.73 m ^2^ /year. The comparison between the fast and the remaining progressor patients showed that the former ones were younger [70 (68–75) vs. 73 (69–79) years; p = 0.01], had higher prevalence of diabetic nephropathy (45 vs. 28%, p = 0.03) and higher eGFR 1 [35.5 (25.5–45.5) vs. 28.2 (19.4–37.3) mL/min/1.73 m^2^; p = 0.001]. The other variables were similar in both groups.

## Discussion

In the present single center retrospective observational study, regardless the equation used to estimate GFR, almost 60% of the elderly patients showed a decline in kidney function over a median of two years of follow-up. CKD progression was related to younger age, diabetic nephropathy, higher serum phosphorus and proteinuria.

It has been reported a rate of CKD progression varying from 13% to 70% [8, 12, 13]. Such wide variability might be partially explained by different equation, characteristics of the studied populations and the different criteria used across studies to define CKD progression. Although the calculation of GFR by CKD-EPI for elderly people has been questioned, studies that compared CKD-EPI’s accuracy with equations like BIS-1 and BIS– 2, developed specifically for elderly, there were no difference [4, 5]. Confirming this, in the present study, the CKD progression was calculated by CKD-EPI and BIS-1 was similar. Other reason that explain the high variation of CPK progression prevalence are the different criteria uses in the definition of progression. In the last years, CKD progression has been defined by reduction in 25–50% of GFR [14], change of CKD category [2], or the mean annual change of GFR over follow-up time, expressed in mL/min/year [9, 10]. Arora et al [10], in an elderly cohort, showed that less than fifty percent present CKD progression. Comparing our results, this less prevalence could be related with the definition of CKD progression (- 1 mL/min/1.73 m ^2^ /year) used in the study, once we and other authors [13] utilized a valor of < 0 mL/min/1.73 m ^2^ /year and found higher rates of progression. Unexpectedly, 43% of the patients presented some increase of eGFR during the study. The reasons why there was an improvement in the kidney function remain to be clarified. One could hypothesize that the implementation of the treatment could have had a beneficial impact in the eGFR. Moreover, some of the patients could have experienced an acute renal insult before the admission in the program. Of note, similar to our findings some authors also observed in prospective studies that 30% and 48% of the patients maintained or improved kidney function during the follow up [12, 13].

Several factors could be related to CKD progression, such as baseline eGFR, age, presence of proteinuria, and diabetes among others. Older age has been associated with the occurrence and higher progression of CKD [10, 11, 15, 16]. In a 3.322 adults patients cohort, 13% of the patients that declined kidney function 77% were elderly [8]. However, like ours, other studies [8, 13, 17] showed that the eGFR slope and its magnitude were higher in younger patients. A possible explanation for that, suggested by O´Hare et al, [18] is the competing risk for death over end renal stage kidney disease present in the elderly population. Besides age, the category of CKD influences on the disease progression. Elderly cohorts or predominantly elderly, the prevalence of CKD progression was higher as less as GFR of baseline with rates until 70% in patients with GFR < 30mL/min/1.73m^2^ [9, 12]. A meta-analysis that included 2 million participants, found that the lower eGFR at baseline, the higher magnitude of CKD progression [19]. This fact was confirmed when evaluated just elderly patients [9]. Differently, on this study, the GFR on baseline was similar in the progression and non progression groups. Unlike expected, fast progressors patients present a higher GFR at baseline. This fact could be observed for other authors [8].

The presence of proteinuria, the major marker of kidney damage, is the best-known risk factor for CKD progression. Several studies observed that proteinuria was associated with a faster rate of kidney function decline and achievement of ESRD [13, 19–21]. Recently, Arora et al [10], in an elderly cohort, similar to ours, showed that proteinuria was independently associated with increased eGFR slope.

Another important risk for CKD progression and faster decline of renal function is the presence of diabetes, in general [21, 22] and elderly population [9, 10]. Of note, in the present study, a higher prevalence of diabetic nephropathy in the progressor as well as in faster progressor patients was observed. However, in the multiple model analysis the presence of diabetic nephropathy was not associated to CKD progression probably due to its close relationship with proteinuria. Other factor involved in CKD progression is the calcium-phosphorus metabolism disorders. Several studies have shown an association between high serum phosphorus and risk of CKD progression [13, 23]. In our study, higher serum phosphorus was observed in the progressor group. Experimental study demonstrated that an increase phosphate excretion per nephron was associated with tubule-interstitial lesions including tubular atrophy and dilatation [24]. Moreover, high phosphorus contributes to the formation of calciprotein (cytotoxic nanoparticles of calcium-phosphate crystal and mineral binding proteins) that cause chronic inflammation and tubular damage [25]. Although based in unclear mechanisms, increased FGF-23 and decreased klotho expression, due to phosphorus overload [26], seem also to be related to CKD progression [23].

Some limitations of this study should be address: a single center and retrospective study, a relatively small follow up and proteinuria evaluated by spot urine sample. Despite that, using the two currently recommended equations for estimate GFR, this study was able to identify CKD progression in the majority of elderly patients.

In conclusion, CKD progression was observed in the majority of elderly CKD patients and proteinuria was the most important factor associated to kidney function decline in this population.
